# Supplementary material for: Needs assessment and preparedness of the primary health care network for scaling-up preventive tuberculosis treatment in 5 Brazilian capitals
Source: PLoS One. 2025 Jun 27;20(6):e0326428. doi: 10.1371/journal.pone.0326428 (PMC12204585; doi:10.1371/journal.pone.0326428)
Supplement: S1 File — (PDF) [file pone.0326428.s001.pdf]

Survey instrument used for data collection

1) Name of the town.

2) The health facility offers tuberculin skin testing?
Yes
No

3) Does the facility have a health care worker for tuberculin skin testing reading?
Yes
No

4) Does the facility have a fridge for purified protein derivative storage?
Yes
No

5) Does the facility conduct blood withdrawal for interferon-gamma release?
Yes
No

6) What is the distance (km) for interferon-gamma release assay lab?

7) What is the mean time, in days, for interferon-gamma release assay results?

8) Does unit have a chest X-ray equipment?
Yes
No

9) Has 3 months of weekly doses of rifapentine and isoniazid been the preferred regimen for adults?
Yes
No
