# Supplementary material for: Needs assessment and preparedness of the primary health care network for scaling-up preventive tuberculosis treatment in 5 Brazilian capitals
Source: PLoS One. 2025 Jun 27;20(6):e0326428. doi: 10.1371/journal.pone.0326428 (PMC12204585; doi:10.1371/journal.pone.0326428)
Supplement: Attachment — Submitted filename: response_to_reviewers.docx [file pone.0326428.s004.docx]

PONE-D-25-04795

Needs assessment and preparedness of the primary health care network for scaling-up preventive tuberculosis treatment in 5 Brazilian capitals.

Dear Dr. Frederick Quinn

Editor, PLOS ONE

We are pleased to submit for your consideration a revised version of our manuscript entitled Needs assessment and preparedness of the primary health care network for scaling-up preventive tuberculosis treatment in 5 Brazilian capitals. You will find below a point-by-point reply to the reviewers’ comments. We believe that their suggestions were very useful to improve our manuscript and hope this revised version is suitable for publication in Plos One.

All authors have approved this revised version of the manuscript and take full responsibility for all its aspects.

We certify that this manuscript is original, has not been published in whole or in part, and is not under consideration for publication in any other journal, whether in print or electronic format.

We declare that there are no conflicts of interest related to this work.

The funders had no role in study design, data collection and analysis, decision to publish, or preparation of the manuscript.

Dinah Carvalho Cordeiro, on behalf of all authors

Journal Requirements:

When submitting your revision, we need you to address these additional requirements.

Response: *Thank you for reviewing our manuscript and for your suggestions to improve it further.*

1. Please ensure that your manuscript meets PLOS ONE's style requirements, including those for file naming. The PLOS ONE style templates can be found at <https://journals.plos.org/plosone/s/file?id=wjVg/PLOSOne_formatting_sample_main_body.pdf> and <https://journals.plos.org/plosone/s/file?id=ba62/PLOSOne_formatting_sample_title_authors_affiliations.pdf>

*Response: We have revised the formatting of the manuscript to meet PLOS ONE’s requirements.*

2. We note that the grant information you provided in the ‘Funding Information’ and ‘Financial Disclosure’ sections do not match.

When you resubmit, please ensure that you provide the correct grant numbers for the awards you received for your study in the ‘Funding Information’ section.

*Response: We revised.*

3. Thank you for stating the following financial disclosure:
Stop TB Partnership, TB REACH wave 10, project #10429.

Please state what role the funders took in the study. If the funders had no role, please state: "The funders had no role in study design, data collection and analysis, decision to publish, or preparation of the manuscript." If this statement is not correct you must amend it as needed. Please include this amended Role of Funder statement in your cover letter; we will change the online submission form on your behalf.

*Response: We included the text “**The funders had no role in study design, data collection and analysis, decision to publish, or preparation of the manuscript” in the cover letter.*

4. We note that your Data Availability Statement is currently as follows: Todos os dados relevantes estão dentro do manuscrito e de seus arquivos de Informações de Suporte.

Please confirm at this time whether or not your submission contains all raw data required to replicate the results of your study. Authors must share the “minimal data set” for their submission. PLOS defines the minimal data set to consist of the data required to replicate all study findings reported in the article, as well as related metadata and methods (<https://journals.plos.org/plosone/s/data-availability#loc-minimal-data-set-definition>).

For example, authors should submit the following data:

- The values behind the means, standard deviations and other measures reported;

- The values used to build graphs;

- The points extracted from images for analysis.

Authors do not need to submit their entire data set if only a portion of the data was used in the reported study.

If your submission does not contain these data, please either upload them as Supporting Information files or deposit them to a stable, public repository and provide us with the relevant URLs, DOIs, or accession numbers. For a list of recommended repositories, please see <https://journals.plos.org/plosone/s/recommended-repositories>.

If there are ethical or legal restrictions on sharing a de-identified data set, please explain them in detail (e.g., data contain potentially sensitive information, data are owned by a third-party organization, etc.) and who has imposed them (e.g., an ethics committee). Please also provide contact information for a data access committee, ethics committee, or other institutional body to which data requests may be sent. If data are owned by a third party, please indicate how others may request data access.

*Response: We are submitting the database as supplementary information and have requested Rede-TB to host the database as well .*

5. Please review your reference list to ensure that it is complete and correct. If you have cited papers that have been retracted, please include the rationale for doing so in the manuscript text, or remove these references and replace them with relevant current references. Any changes to the reference list should be mentioned in the rebuttal letter that accompanies your revised manuscript. If you need to cite a retracted article, indicate the article’s retracted status in the References list and also include a citation and full reference for the retraction notice.

[Note: HTML markup is below. Please do not edit.

*Response: All references have been reviewed to ensure they follow the Vancouver style, and access links have been verified.*

Reviewers' comments:

Reviewer's Responses to Questions

**Comments to the Author**

1. Is the manuscript technically sound, and do the data support the conclusions?

The manuscript must describe a technically sound piece of scientific research with data that supports the conclusions. Experiments must have been conducted rigorously, with appropriate controls, replication, and sample sizes. The conclusions must be drawn appropriately based on the data presented.

Reviewer #1: Yes

Reviewer #2: Partly

2. Has the statistical analysis been performed appropriately and rigorously?

Reviewer #1: Yes

Reviewer #2: No

3. Have the authors made all data underlying the findings in their manuscript fully available?

The [PLOS Data policy](http://track.editorialmanager.com/CL0/http:%2F%2Fwww.plosone.org%2Fstatic%2Fpolicies.action%23sharing/1/010f0195a6c8e043-f1fee2df-d5de-49a3-bb0b-be9ec98d7802-000000/wOPv0aD8TH3EBL_JMJrd7UtJeDD0y25IlJtQnTuEqVw=202) requires authors to make all data underlying the findings described in their manuscript fully available without restriction, with rare exception (please refer to the Data Availability Statement in the manuscript PDF file). The data should be provided as part of the manuscript or its supporting information, or deposited to a public repository. For example, in addition to summary statistics, the data points behind means, medians and variance measures should be available. If there are restrictions on publicly sharing data—e.g. participant privacy or use of data from a third party—those must be specified.

Reviewer #1: No

Reviewer #2: Yes

4. Is the manuscript presented in an intelligible fashion and written in standard English?

PLOS ONE does not copyedit accepted manuscripts, so the language in submitted articles must be clear, correct, and unambiguous. Any typographical or grammatical errors should be corrected at revision, so please note any specific errors here.

Reviewer #1: Yes

Reviewer #2: Yes

5. Review Comments to the Author

Please use the space provided to explain your answers to the questions above. You may also include additional comments for the author, including concerns about dual publication, research ethics, or publication ethics. (Please upload your review as an attachment if it exceeds 20,000 characters)

Reviewer #1: Very interesting paper over implementation of interventions for a clear PH problem. Please see my suggestions/ remarks for consideration

*Response: Thank you for your encouragemen and for your very useful suggestionst.*

Settings
1. Could it be clarified if the different regions researched are different in looking at urban, rural facilities

*Response: We clarified that the clinics were located in urban áreas (lines 94-96).*

Results

2. Please consider removing everywhere the ‘excluding missing data”. With the nominator and denominator and mention in the methods is already clear that missing data is excluded and now it is sometimes mentioned where there even is no data missing.

*Response: We have excluded.*

3. There are difference seen in the results of the different areas. Can there be more mention of that in the results. Are there differences in rural versus urban?

*Response: We were not able to evaluate if there are differences between rural and urban areas, since the selected clinics were all in urban areas, but we did observe differences between cities. Rio de Janeiro had more professionals qualified and more clinics that offered the tuberculin skin test. This may be due to the fact that some research on latent tuberculosis was conducted in Rio de Janeiro and, at that time, there was intensive training in performing the tuberculin skin test.We clarify this in the results and discussion. We have added that to the text (lines 148-151).*

Discussion

4. I suggest the first paragraph of the discussion be moved to the introduction. It more clearly explains the need to implement these strategies and why this assessment than is so importante

*Response: We have moved as suggested.*

5. There is mention of little personnel skilled to perform TST due to the stringent criteria. I miss the discussion whether this is fair or how this could be dealt with

*Response: We included this information in the manuscript (lines 145-147). Recent changes to national recommendations for training health professionals in tuberculin skin test were published by the Ministry of Health in December 2024. These changes were based on data from the ExpandTPT project and other evidence. The change is now one of the references.*

6. Consider discussing the different aspects looked at separately.

*Response: We have summarized the findings in the first paragraph and have discussed diferente aspects separately.*

7. Please clarify the point regarding stockouts of PPD jeopardizing training

*Response: We have included in the text (lines154-155). We could not use the remaining doses of PPD for training as we saved it for the situations in which it eas most needed. We hope this is now clear.*

8. The point regarding WHO recommendations to offer TB preventive treatment without tests and the following paragraph is not clear. Could authors please clarify what they are recommending for their country

*Response: We have rephrased (lines 166-168).*

9. Consider elaborating in the discussion about the different findings by region. With some clear recommendations

*Response:* *Our recommendations are general, because although Rio de Janeiro has more clinics offering the tuberculin skin test, it still needs to expand the offer of the test. We have includde this in the discussion (lines 148-151).*

Reviewer #2: Title: Needs assessment and preparedness of the primary health care network for scaling-up preventive tuberculosis treatment in 5 Brazilian capitals

The manuscript is well-written and effectively addresses the challenges of scaling up tuberculosis preventive treatment (TPT) in five high-burden Brazilian cities through a descriptive and observational study design, suitable for identifying implementation gaps.

*Response: Thank you for your review and for your suggestions. We believe they have improved the manuscript.*

Methodology: The variation in TPT prescription rates suggests disparities in implementation across cities, possibly due to differences in healthcare infrastructure, resource allocation, or adherence to national guidelines. I wonder if the authors collected other factors that could drive these disparities and develop targeted interventions to ensure equitable TPT access across all high-burden areas.

*Response:* *The main difference between the cities is the availability of tuberculin skin testing. We believe this is due to the intensive training for preventive treatment research in Rio de Janeiro. We have added this discussion in lines 148-151.*

The inclusion of the questionnaire as supplemental material would be valid and enable others to understand the specific questions asked, the structure of the survey, and the context in which responses were gathered.

*Response:* *We have included our questionnaire as supplementary material.*

Statistical analysis is missing, only descriptive analysis was conducted, stratified by city, with percentages calculated excluding missing data.

*Response:* *We did not intend an analytical approach to compare the five cities because the numbers were small; the descriptive analysis was sufficient for our conclusions. We have now included the 95%CI in the text and table.*

Discussion: as a suggestion, some points that can be included in the discussion to make it more robust include 1. consider the nature of the study, limiting the ability to assess trends or long-term intervention 2. the potentially biased self-reported data from clinic directors and the absence of patient perspectives, such as stigma or awareness issues, which could influence TPT uptake.

*Response: Thank you for these suggestions. We have included these points as limitations: “*Our study has limitations. Self-reported data by clinic staff may potentially generate bias. Thus, true data may be still worse. Another limitation is the absence of health system users’ perspective that could influence adherence to testing and tuberculosis preventive treatment uptake”.

Table 1: please correct alignment of the row 3 (Health…), 5 (Blood…) and 9 (3 months…)

*Response: We have corrected.*

6. PLOS authors have the option to publish the peer review history of their article ([what does this mean?](https://track.editorialmanager.com/CL0/https:%2F%2Fjournals.plos.org%2Fplosone%2Fs%2Feditorial-and-peer-review-process%23loc-peer-review-history/1/010f0195a6c8e043-f1fee2df-d5de-49a3-bb0b-be9ec98d7802-000000/SOKF6RFaHzdUaXMHdxSkjxC3XRoI3qX6F0THrImyyRI=202)). If published, this will include your full peer review and any attached files.


If you choose “no”, your identity will remain anonymous but your review may still be made public.

**Do you want your identity to be public for this peer review?** For information about this choice, including consent withdrawal, please see our [Privacy Policy](https://track.editorialmanager.com/CL0/https:%2F%2Fwww.plos.org%2Fprivacy-policy/1/010f0195a6c8e043-f1fee2df-d5de-49a3-bb0b-be9ec98d7802-000000/jweU8JWdzjyxZg9S4ylK58LLfyDvdHgknIgByYcr74M=202).

Reviewer #1: No

Reviewer #2: No
